# BDNF Activates mTOR to Regulate GluR1 Expression Required for Memory Formation

**DOI:** 10.1371/journal.pone.0006007

**Published:** 2009-06-23

**Authors:** Leandro Slipczuk, Pedro Bekinschtein, Cynthia Katche, Martín Cammarota, Iván Izquierdo, Jorge H. Medina

**Affiliations:** 1 Instituto de Biología Celular y Neurociencias, Facultad de Medicina, Universidad de Buenos Aires (UBA), Buenos Aires, Argentina; 2 Departamento de Fisiología, Facultad de Medicina, Universidad de Buenos Aires (UBA), Buenos Aires, Argentina; 3 Centro de Memoria, Instituto de Pesquisas Biomedicas, Pontifícia Universidade Católica do Rio Grande do Sul (PUCRS), Porto Alegre, Brasil; INSERM U862, France

## Abstract

**Background:**

The mammalian target of Rapamycin (mTOR) kinase plays a key role in translational control of a subset of mRNAs through regulation of its initiation step. In neurons, mTOR is present at the synaptic region, where it modulates the activity-dependent expression of locally-translated proteins independently of mRNA synthesis. Indeed, mTOR is necessary for different forms of synaptic plasticity and long-term memory (LTM) formation. However, little is known about the time course of mTOR activation and the extracellular signals governing this process or the identity of the proteins whose translation is regulated by this kinase, during mnemonic processing.

**Methodology/Principal Findings:**

Here we show that consolidation of inhibitory avoidance (IA) LTM entails mTOR activation in the dorsal hippocampus at the moment of and 3 h after training and is associated with a rapid and rapamycin-sensitive increase in AMPA receptor GluR1 subunit expression, which was also blocked by intra-hippocampal delivery of GluR1 antisense oligonucleotides (ASO). In addition, we found that pre- or post-training administration of function-blocking anti-BDNF antibodies into dorsal CA1 hampered IA LTM retention, abolished the learning-induced biphasic activation of mTOR and its readout, p70S6K and blocked GluR1 expression, indicating that BDNF is an upstream factor controlling mTOR signaling during fear-memory consolidation. Interestingly, BDNF ASO hindered LTM retention only when given into dorsal CA1 1 h after but not 2 h before training, suggesting that BDNF controls the biphasic requirement of mTOR during LTM consolidation through different mechanisms: an early one involving BDNF already available at the moment of training, and a late one, happening around 3 h post-training that needs *de novo* synthesis of this neurotrophin.

**Conclusions/Significance:**

In conclusion, our findings demonstrate that: 1) mTOR-mediated mRNA translation is required for memory consolidation during at least two restricted time windows; 2) this kinase acts downstream BDNF in the hippocampus and; 3) it controls the increase of synaptic GluR1 necessary for memory consolidation.

## Introduction

Translational control in eukaryotic cells is critical for gene regulation during nutrient deprivation and stress, development and differentiation, nervous system function, aging, and disease [1]. A prevailing view indicates that long-lasting forms of synaptic plasticity and memory require new protein synthesis across multiple experimental preparations and species. These plasticity-related proteins are supposed to stabilize synaptic reinforcement that occurs after a learning event [2]–[5]. However, the questions of which proteins are translated during memory formation and which are the signals triggered by the learning experience to regulate such translation remain unanswered.

mTOR is a high molecular-weight serine-threonine protein kinase that modulates cell growth, proliferation and synaptic plasticity via the regulation of protein synthesis [6] specifically controlling the translation of a subset of mRNAs that contain extensive secondary structure at their 5′ UTR or an oligopyrimidine tract in their 5′ end (TOP mRNAs) [7]. This kinase can be activated by different extracellular signals and regulates protein synthesis at the initiation level mainly through the phosphorylation of at least two downstream targets, p70S6 kinase (p70S6K) and eukaryotic initiation factor 4E-binding proteins (4E-BPs, see for references, [8]). In neurons, mTOR is present at the synaptic region where it modulates the synthesis of locally-translated proteins, is upregulated in an activity-dependent manner and is critical for different forms of synaptic plasticity, including long-term potentiation (LTP) [9], [10]. In addition, several studies have implicated mTOR signaling in memory processing.

Rapamycin is a specific inhibitor of mTOR function that prevents p70S6K and 4E-BPs phosphorylation thus interfering with the initiation of translation [11] of a subset of mRNAs rather than general translation [12]. When administered around training, rapamycin blocks LTM formation in a number of learning tasks [13]–[17]. However, little is known about the extracellular signals triggered by training that are essential to activate mTOR for the regulation of protein synthesis during memory consolidation. Brain-derived neurotrophic factor (BDNF) is a member of the family of neurotrophins intimately implicated in synaptic plasticity and memory. BDNF is capable of inducing the late phase of long-term potentiation even in the absence of electrical stimulation and is not only necessary but sufficient for LTP and persistence of LTM storage in the hippocampus [18], [19]. Moreover, this protein is required for memory formation in many learning tasks [15], [20]. Given that BDNF induces rapamycin-sensitive synaptic potentiation [9] and regulates translation of 5′ TOP mRNA encoded proteins at dendrites through an mTOR-dependent pathway [21], we and others hypothesized that this neurotrophin could control mTOR activation state during memory processing [22].

It has been previously proposed that memory consolidation and persistence may rely on multiple waves of protein synthesis [23], [24]. For instance, a second wave of sensitivity to anisomycin, a well-known, general protein synthesis inhibitor, has been found 3–9 h after training in a number of learning tasks [25]–[29]. Therefore, the aims of the present study were firstly to examine if a single or multiple mTOR-dependent phase(s) is/are involved in the regulation of protein synthesis required for LTM formation, secondly to determine whether BDNF plays a role in mTOR-dependent regulation of LTM formation and finally to identify plasticity-related proteins required for memory formation regulated by this pathway.

## Results

### Two Time Windows of mTOR Activation Are Required for Consolidation of Inhibitory Avoidance LTM

To investigate the effect of learning on the activation of mTOR and its downstream effector p70S6K we used a one-trial step-down inhibitory avoidance paradigm (IA). This fear-motivated associative learning task is hippocampus-dependent and acquired in a single and brief training session [30]–[35], which makes it suitable for investigating time-dependent mechanisms initiated by training [15], [36] without the possible interference of retrieval of the learned response that occurs in multi-trial tasks [5], [37].

**Figure 1:**
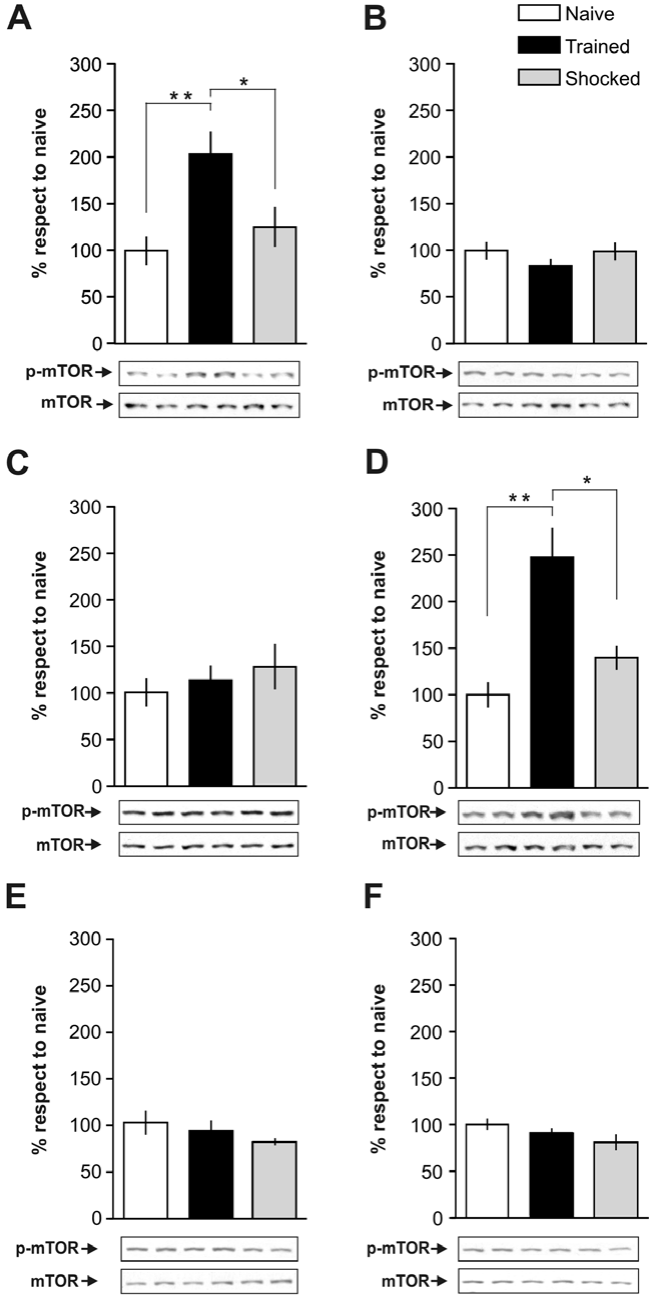
IA Training Is Associated with Two Time Windows of mTOR Activation in the Hippocampus. Bars represent the mean p-mTOR/mTOR ratio of trained (black) or shocked (gray) groups respect to naïve (white) group, sacrificed immediately (A), 15 min (B), 1 h (C), 3 h (D), 9 h (E) or 12 h (F) after IA training. Data are expressed as means±SEM of p-mTOR/mTOR ratio; *p<0.05, **p<0.01. Representative blots of phosphorylated and total protein levels of mTOR are shown in the lower panels. n = 5–6 per group for each experiment.

Confirming and extending previous findings [15], we found that IA training is associated with mTOR activation in the dorsal hippocampus (Figure 1). Immunoblot analysis with an antibody that detects mTOR only when phosphorylated at serine 2448 (p-mTOR), i.e., when active, revealed two peaks of increased p-mTOR immunoreactivity in the dorsal hippocampus of trained rats sacrificed immediately (0 h) or 3 h after training (0 h: +104% respect to naïve, p<0.001, n = 5; 3 h: +148% respect to naïve, p<0.001, n = 5). On the other hand, no changes in p-mTOR immunoreactivity were found 15 min, 1 h, 9 h or 12 h after training. Moreover, the total amount of mTOR in trained animals remained unaltered when compared to naïve or shocked controls. Importantly, the IA training-induced activation of mTOR is learning-specific since no changes in p-mTOR levels were found in shocked control animals.

**Figure 2:**
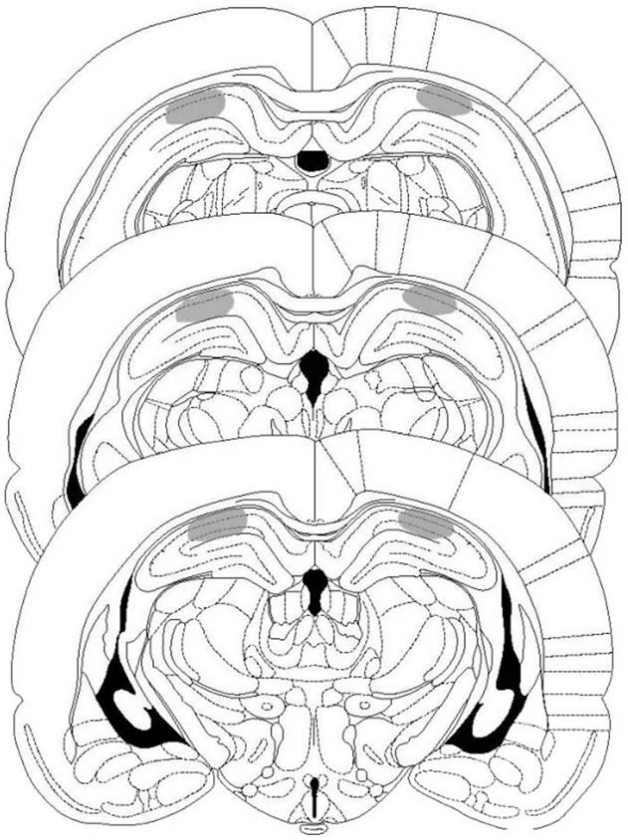
Cannulae Placements and Drug Infusions. Schematic representations of rat brain sections at three rostrocaudal planes (−3.80, −4.30, and −4.80 from bregma) taken from the atlas of Paxinos and Watson, showing, in stippling, the extension of the area reached by the infusions in the dorsal hippocampus. Reprinted from The Rat Brain in Stereotaxic Coordinates by Paxinos and Watson, pages 33, 35, and 37, Academic Press (1997), with permission from Elsevier.

**Figure 3:**
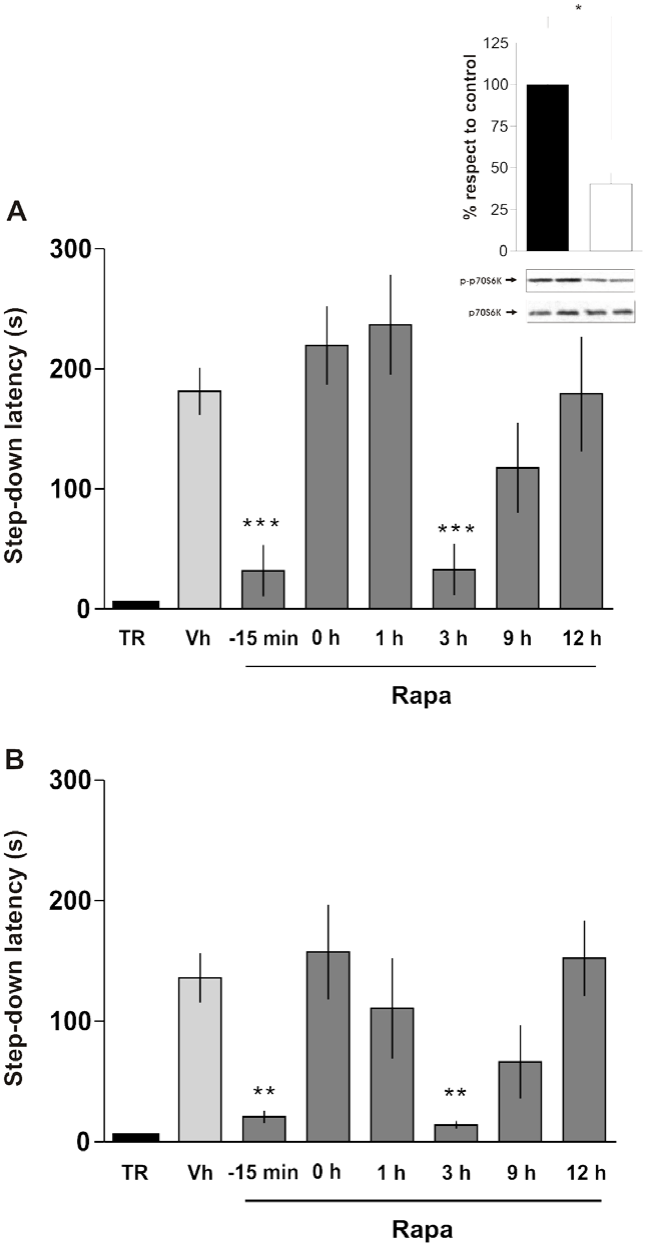
mTOR Signaling Is Required for IA Memory Consolidation During Two Restricted Time Windows After Training. Data are expressed as mean (±SEM) of training (TR, black bars) or test session step-down latency at 24 h (A) or 7 days (B) after IA training. Animals were infused intra-CA1 of the dorsal hippocampus with vehicle (Vh, light grey bars) or rapamycin (4.3 pg/side, dark grey bars), 15 min before or 0, 1, 3, 9, and 12 h after training. Inlet: Rats were infused into the dorsal hippocampus with rapamycin (4.3 pg/side) or vehicle and sacrificed 15 min after drug infusion for analysis of p70S6K activation in the dorsal hippocampus by Western blot. Bars represent the p-p70S6K/p70S6K ratio of rapamycin (white) respect to vehicle (black) treated rats. *p<0.05, **p<0.01, ***p<0.001 vs. Vh for each time point, n = 8–10 per group for each experiment in A and B and n = 5 per group in the inlet.

To determine the time course of mTOR activity requirement for IA LTM formation we infused rapamycin, a highly specific inhibitor of mTOR [38]–[42], into the CA1 region of the dorsal hippocampus (Figure 2) 15 min before or at different times after training. As can be seen in Figure 3A, rapamycin impaired LTM retention when administered 15 min before or 3 h after training (p<0.001, n = 8–10). However, no effect was seen when rapamycin was infused at 0 h, 1 h, 9 h, or 12 h after training, indicating that the amnesia induced by this drug is not attributable to impairment of retrieval or to nonspecific behavioral effects. The impairment in LTM retention 24 h after training produced by rapamycin was also observed in a different group of IA trained rats tested 7 days post-training (Figure 3B), indicating that the amnesic effect of rapamycin is long-lasting.

p70S6K is activated by phosphorylation at Thr-389 solely via mTOR and it is widely used as a readout of mTOR activity [13]–[15], [21], [43]. Thus, we used phospho-p70S6K (p-p70S6K) immunoblot to determine the effectiveness of rapamycin infusions (Figure 3A inlet). At the dose used in the pharmacological experiments presented above (4.3 pg/side), the intra-CA1 infusion of rapamycin inhibited hippocampal p70S6K phosphorylation by more than 60% (p<0.05, n = 5). Indeed, when infused into dorsal CA1 15 min before IA training, rapamycin prevented the learning-induced phosphorylation of p70S6K observed at 15 min post-training (Vehicle = 131.7±3.12, rapamycin = 92.68±4.88 vs naïve animals p<0.001 in one-way ANOVA, n = 5). Moreover, when administered 2∶45 h after the training session it blocked the IA-induced increase in hippocampal p70S6K phosphorylation levels 3 h post-training (Vehicle = 141.4±34.03, rapamycin = 74.51±21.96 vs naïve animals p<0.01. One-way Anova, n = 6).

Together, these findings indicate that the amnesic effect of rapamycin is long-lasting and that there are, at least, two time windows during which rapamycin is able to induce amnesia for IA memory: one around the time of training and the other one 3 h later. These two periods of sensitivity to rapamycin overlap with the two peaks of increased hippocampal p-mTOR levels observed after IA training (Figure 1), indicating that learning of the IA response requires mTOR activity around the time of training and once again 3 h later.

### BDNF Is an Up-Stream Activator of mTOR During Memory Formation

Having determined the existence of two post-training windows of mTOR activity that are necessary for IA memory consolidation, we set to identify the extracellular signals that could be driving mTOR biphasic activation necessary for memory processing.

Given that BDNF regulates local protein synthesis in dendrites [21], [43]–[45] through an mTOR-dependent pathway [21] and also induces LTP that is blocked by rapamycin [9], we examined if BDNF could be triggering mTOR activation during IA training.

**Figure 4:**
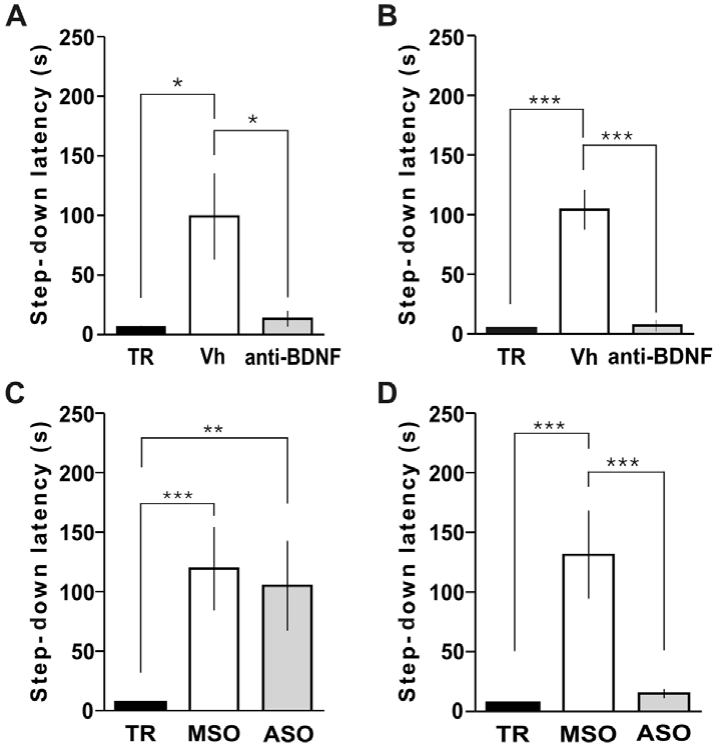
Different Requirement of BDNF Activity and Synthesis During the Two Time Windows of Memory Consolidation. Data are expressed as mean (±SEM) of training (TR, black bars) or test session step-down latency at 24 h after IA training. Animals were infused intra-CA1 of the dorsal hippocampus with vehicle (Vh, white bars) or function-blocking anti-BDNF antibodies (1 µg/µl, grey bars) 15 min before (A) or 3 h (B) after training. Animals were infused intra-CA1 of the dorsal hippocampus with BDNF MSO (white bar) or BDNF ASO (2 nmol/µl, 1 µg/side, grey bar), 2 h before (C) or 1 h (D) after IA training. *p<0.05, **p<0.01, ***p<0.001 vs Vh (A, B) or MSO (C, D), n = 10 per group for each experiment.

Rats infused with function-blocking anti-BDNF antibodies into dorsal CA1 15 min before or 3 h after IA training were amnesic when tested 24 h post-training (Figure 4A, 4B), suggesting a role for BDNF in mTOR pathway. Albeit function-blocking anti-BDNF antibodies block IA memory consolidation when administered pre-training, they have no effect when injected immediately after training [36]. Moreover, it is known that BNDF vesicles are rapidly released in an activity dependent manner [20]. For that reasons we wondered if the two time windows described above were different in their need for new BDNF synthesis. We have previously shown that BDNF oligonucleotides (BDNF ASO) block learning-induced BDNF synthesis in the hippocampus 2 h after injection without lowering basal BDNF levels [24]. Therefore, we injected BDNF ASO and BDNF scrambled missense oligonucleotides (BDNF MSO) into the hippocampus 2 h before or 1 h after training to inhibit BDNF synthesis around or 3 h after IA training, respectively. We found that even though BDNF synthesis was necessary for memory consolidation during the second time window (about 3 h after training, Figure 4D), it was not required at the moment of training (Figure 4C). A feasible explanation could be that stored, previously synthesized BDNF is released immediately post-training during the first window, whereas new BDNF protein has to be synthesized during the second time window, around 3 h post-training.

**Figure 5:**
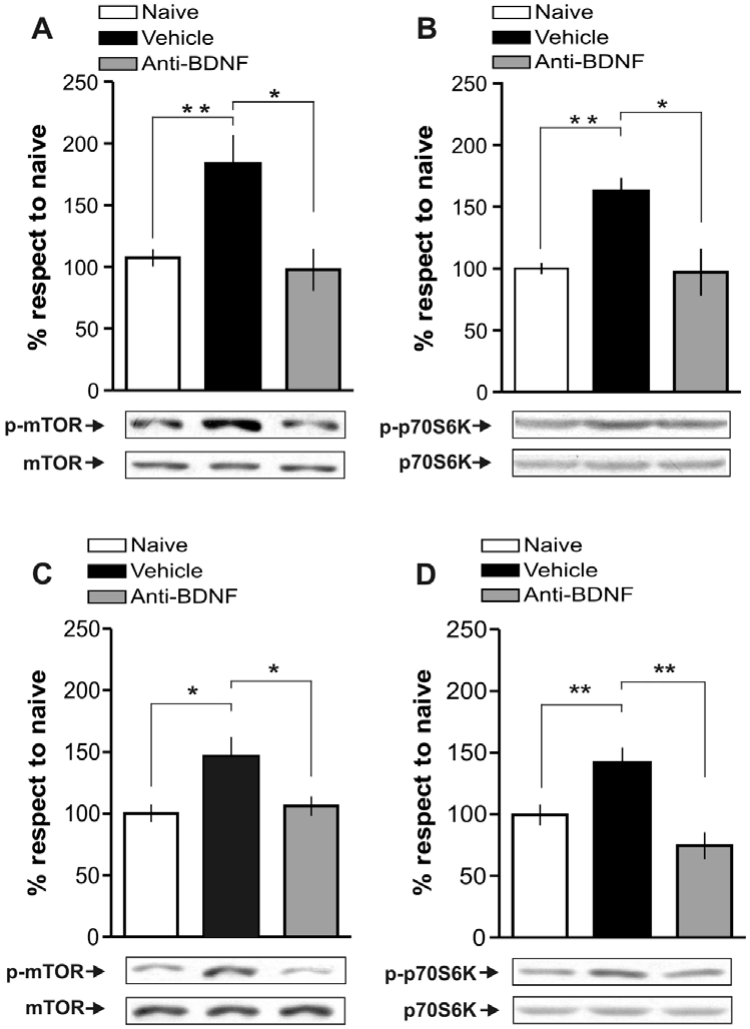
BDNF Triggers mTOR Activation in the Hippocampus During IA Training and 3 h Thereafter. Bars represent mean p-mTOR/mTOR (A and C) or p-p70S6K/p70S6K ratio (B and D) of rats infused with vehicle (black), function-blocking anti-BDNF antibodies (0.5 µg/side; grey), respect to the naïve group (white). Intra-CA1 infusion of function-blocking anti-BDNF antibodies 15 min before IA training prevents mTOR (A) and p70S6K (B) activation immediately or 15 min after training respectively and infusion 2∶45 h after training prevents mTOR (C) and p70S6K (D) activation 3 h after training. Representative blots of phosphorylated and total protein levels of mTOR and p70S6K are shown in the lower panels. *p<0.05, **p<0.01, n = 5–6 per group for each experiment.

Subsequently we wanted to elucidate whether BDNF was the upstream activator of mTOR during memory consolidation. To begin with, we decided to focus on the first window. We found that blockade of BDNF with function-blocking anti-BDNF antibodies delivered into dorsal CA1 15 min before training abolished the IA learning-induced increase in mTOR (Figure 5A) and p70S6K (Figure 5B) phosphorylation. These findings indicate that endogenous BDNF activates the hippocampal mTOR signaling cascade immediately after training.

Given that the first wave of protein synthesis, which occurs around the time of training, is the best studied regarding its functional role on memory formation, whereas the second wave of protein synthesis is still not well understood [46], we decided to characterize the second window as well.

We found that injection of function-blocking anti-BDNF antibodies 2∶45 h after training hindered the IA learning-induced increase in mTOR (Figure 5C) and p70S6K (Figure 5D) phosphorylation that takes place 3 h after training. These results show that BDNF is the extracellular signal triggering mTOR activation 3 h after training.

### Activation of the BDNF/mTOR Pathway Regulates GluR1 Translation Necessary for Memory Formation

**Figure 6:**
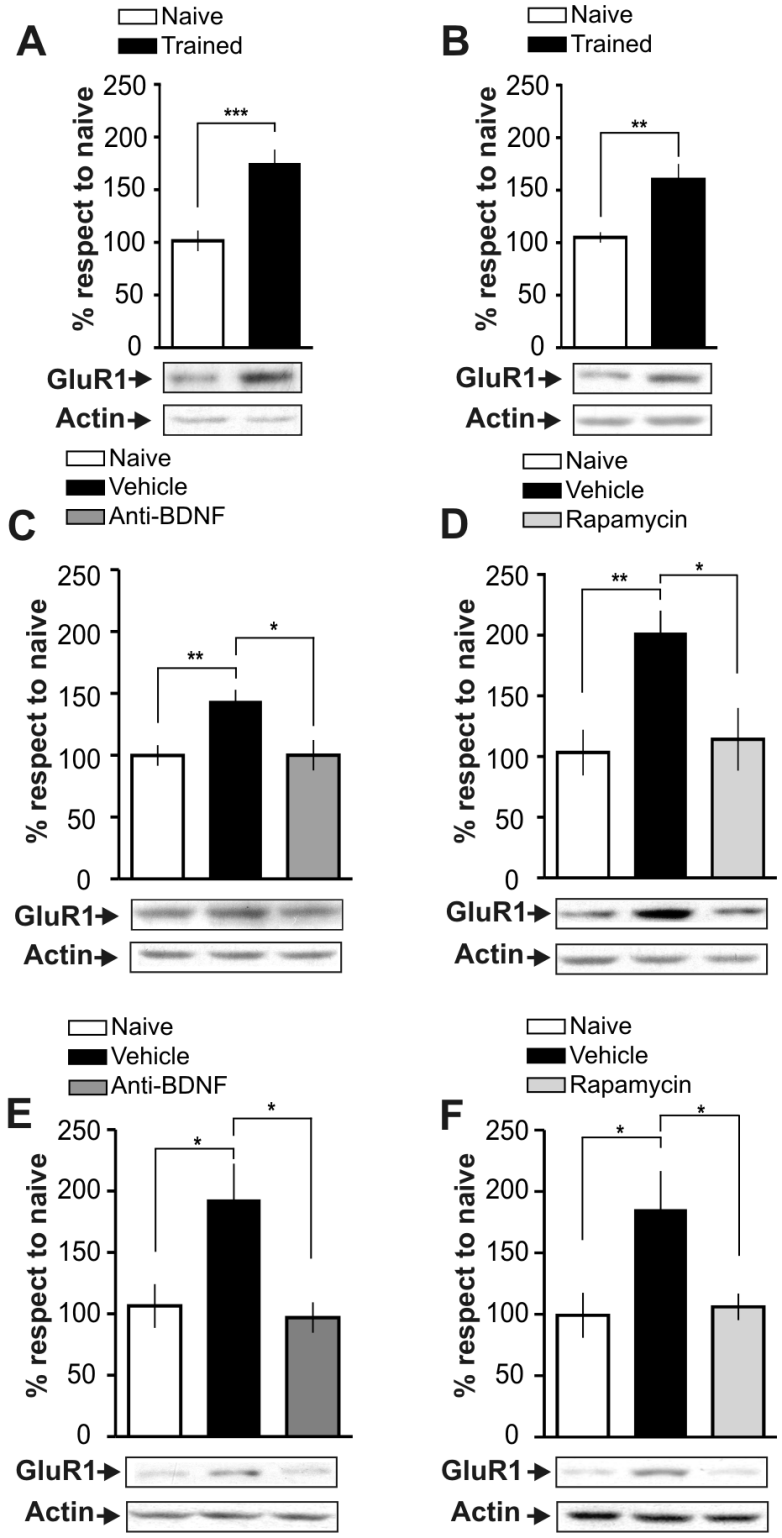
IA Training Induces GluR1 Expression in Hippocampal Synaptic Plasma Membranes-Enriched Fractions Through BDNF/mTOR Pathway. Bars represent the mean GluR1/actin ratio from synaptic plasma membranes-enriched fractions obtained from samples of the dorsal hippocampus of trained (black) respect to naïve (white) rats 15 min (A) or 3 h after IA training (B). Rats infused intra-CA1 of the dorsal hippocampus 15 min before and sacrificed 15 min after training (B and C) or 2∶45 h and sacrificed 3 h both after training (E and F) with vehicle (black) or function-blocking anti-BDNF antibodies (0.5 µg/side; dark grey) (C and E) or rapamycin (4.3 pg/side; light grey) (D and F). Representative blots of total GluR1 protein levels and actin are shown in the lower panels *p<0.05, **p<0.01, ***p<0.001, n = 5–7 per group.

What proteins relevant for memory are regulated by the BDNF/mTOR pathway at the translational level? A likely candidate is the GluR1 subunit of α-amino-3-hydroxy-5-methyl-4-isoxazolepropionic acid (AMPA) receptors. It is known that BDNF enhances the local translation of different glutamate receptor subunits, particularly the GluR1 subunit of AMPA receptor [47]–[49] through activation of mTOR [48] and that BDNF is stored in and released from glutamatergic hippocampal neurons [50]. Therefore, we determined whether IA training increases the expression of GluR1 protein levels in the dorsal hippocampus. Extending previous findings [32], [33], we found that IA training resulted in an increase in the expression of GluR1 protein in a subcellular fraction enriched in synaptic plasma membranes (P2) isolated from the dorsal hippocampus 15 min or 3 h after training (Figure 6A, +75±9 percent respect to naïve animals p<0.001 n = 7; Figure 6B, +55±11 percent respect to naïve animals p<0.01 n = 5). Furthermore this change appears to be specific for the hippocampus, as no changes were found in the amygdala (15 min: TR = 105.5±7.8 respect to naïve p>0.05, n = 5 per group; 3 h: TR = 110.4±9.1 respect to naïve p>0.05. Student's *t* test, n = 5 per group). To determine whether the BDNF/mTOR pathway regulates these increases in GluR1 protein in synaptic plasma membranes, we injected into CA1 region of the dorsal hippocampus function-blocking anti-BDNF antibodies or rapamycin 15 min before training and sacrificed the animals 15 min after training. Both function-blocking anti-BDNF antibodies (Figure 6C) and rapamycin (Figure 6D) hindered the learning-induced increase in GluR1. Furthermore, function-blocking anti-BDNF antibodies (Figure 6E) or rapamycin (Figure 6F) infused 2∶45 after training abolished the increase in GluR1 expression occurring 3 h after training. These experiments indicate that the BDNF/mTOR signaling pathway controls the learning-induced increase in GluR1 15 min or 3 h after training.

**Figure 7:**
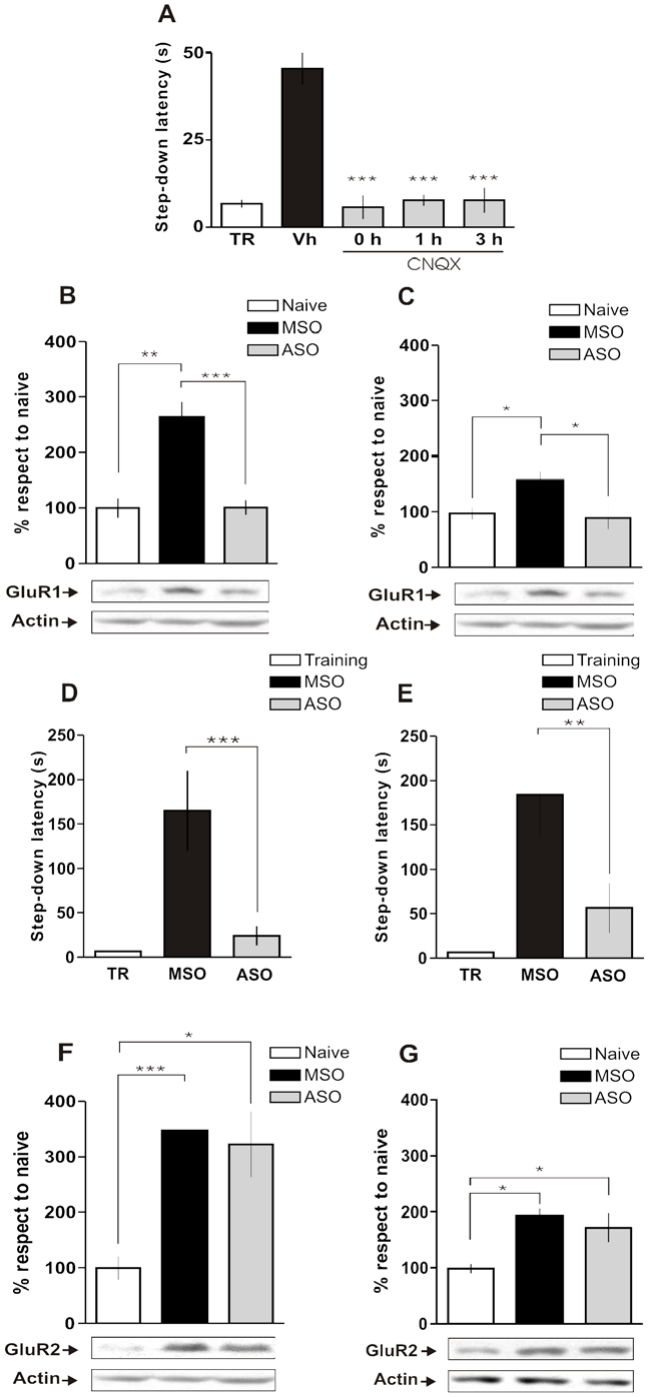
GluR1 Translation Is Required for IA Memory Consolidation During Training and 3 h Thereafter. (A) Animals were injected with Vehicle (black bar) or CNQX immediately, 1 h or 3 h after IA training (grey bars). Data are expressed as mean (±SEM) of training (TR, white bars) or test session step-down latency at 24 h after IA training. (B and C) Bars represent the mean GluR1/actin ratio from synaptic plasma membranes-enriched fractions obtained from samples of the dorsal hippocampus of animals trained in IA and injected with GluR1 ASO 2 h pre-TR (grey bar) or MSO (black bar) and sacrificed 15 min after TR (B) or injected with GluR1 ASO 1 h after TR or MSO and sacrificed 3 h after TR (C). (D and E) Animals were injected with MSO (black bar) or GluR1 ASO (grey bar) 2 h pre-TR (D) or 1 h after TR (E). Data are expressed as mean (±SEM) of training (TR, white bars) or test session step-down latency at 24 h after IA training. (F and G) Bars represent the mean GluR2/actin ratio from synaptic plasma membranes-enriched fractions obtained from samples of the dorsal hippocampus of animals trained in IA and injected with GluR1 ASO 2 h pre-TR (grey bar) or MSO (black bar) and sacrificed 15 min after TR (B) or injected with GluR1 ASO 1 h after TR or MSO and sacrificed 3 h after TR (C). *p<0.05, **p<0.01, ***p<0.001, n = 10–14 per group for each experiment in figures A, D and E; n = 5 per group for each experiment in figures B, C, F and G.

In order to elucidate whether GluR1 is necessary for memory consolidation at the same time points during which the BDNF/mTOR pathway is regulating its translation we infused the AMPA receptor antagonist CNQX into the dorsal hippocampus immediately, 1 h or 3 h after IA training and analyzed its effect on retention 24 h later. As seen in Fig. 7A, CNQX hampered IA LTM consolidation at every post-training time point analyzed. Then, we blocked GluR1 translation during training or 3 h thereafter by injecting GluR1 antisense oligonucleotides (GluR1 ASO) or GluR1 scrambled missense oligonucleotides (GluR1 MSO) into CA1 region of the dorsal hippocampus 2 h before or 1 h after IA training. Initially we observed that administering GluR1 ASO 2 h before training abolished training-induced GluR1 translation 15 min after training (Fig. 7B) and GluR1 ASO injected 1 h after training hindered training induced GluR1 translation 3 h after training (Fig. 7C). We then injected GluR1 ASO or GluR1 MSO at the time points aforementioned and observed that inhibiting GluR1 translation at any of both time points caused amnesia when the animals were tested 24 h after training (Figure 7D, E). Moreover, the effect was specific for *GluR1* mRNA translation as we found no effect of GluR1 ASO on the learning-induced increase in GluR2 protein levels measured in the same membrane preparation (Figure 7F, G). GluR1 scramble missense oligonucleotides did not affect GluR1 levels or memory retention at any post-training time analysed (Figure 7B, C, D, E). These results show for the first time, to our knowledge, that GluR1 translation is necessary around training and again 3 h later for memory consolidation.

As a conclusion, our results show that BDNF is the upstream activator of mTOR around training and 3 h after training to regulate GluR1 translation in hippocampal synaptic plasma membranes during IA training memory consolidation.

## Discussion

Despite a few theoretical interpretations against it [51], [52], the view that macromolecular synthesis is the key step in LTM formation has been gaining more and more evidence during the past 25 years [28], [53]. However, little is known about the extracellular signals that trigger the synthesis of specific and essential proteins involved in the creation of LTMs. Recent experiments show that at least some of the proteins involved in plasticity and memory are synthesized at the synapse from pre-existing mRNAs [54] and mTOR signaling has been found to regulate this local translation [21], [48].

The main findings of the present study are: (1) a biphasic activation of hippocampal mTOR signaling is associated with IA training and is required for its memory formation; (2) activation of the mTOR cascade in the dorsal hippocampus is initiated by BDNF; (3) previously synthesized BDNF is rapidly released immediately after IA training whereas around 3 h after training, new synthesis of BDNF protein is needed for LTM formation; (4) learning related BDNF/mTOR cascade activation after training induces GluR1 expression in hippocampal synaptic plasma membranes; and (5) GluR1 translation during training or 3 h later is required for IA memory consolidation.

We demonstrated the existence of two time windows, one around training and the other 3 h thereafter, during which a specific inhibitor of mTOR activation infused into CA1 produced clear-cut deficits in LTM for a one-trial IA task. These two time periods parallel those observed when the broad range protein synthesis inhibitor anisomycin is used [27], [28], and they agree with previous findings demonstrating the existence of two critical periods of sensitivity of different memories to protein synthesis inhibitors [5], [25]–[29], [55]. In addition, closely related events up- or downstream to protein synthesis also exhibited biphasic activity after training [26], [31], [46], [56]–[59]. Given that the magnitude of the amnesic effect seen with rapamycin is quite similar to that found with anisomycin [28] and that rapamycin decreases protein synthesis only by 10–15% instead of 70–95% as seen with anisomycin [13], [60], the subset of transcripts whose translation is affected by rapamycin seems to be critical for memory formation. These results support the hypothesis that memory consolidation is not a continuous process, but it rather relies on multiple and recurrent waves of protein synthesis to reinforce synaptic connections or to grow new ones [23]. These phases of protein synthesis might have the same or different molecular signatures [28]. It is widely accepted that rapamycin is a highly specific inhibitor of mTOR. This is mainly due to the fact that for rapamycin to be active biologically, it must form a ternary complex with mTOR and FKBP12 (FK506-binding protein 12 kDa), a small cytosolic protein receptor. Rapamycin binds to a specific domain of mTOR and FKBP12 to form a sandwich-like structure that confers an unusually high specificity for rapamycin [61]. However, we cannot totally rule out the possibility that rapamycin may affect other molecular targets.

In considering the role of local protein synthesis in synaptic plasticity underlying memory processing [21], [62], [63], the present results raise several questions: What are the upstream extracellular signals that mediate activation of mTOR signaling required for memory formation? Which are the protein products that are expressed during these waves of translation required for LTM formation? We began to answer these questions by examining whether BDNF triggers the activation of mTOR induced by IA training.

BDNF exerts diverse roles in regulating neuronal structure and function [64], [65]. In particular, it appears to be critical for synaptic plasticity and memory processing in the adult brain [24], [36], [65], [66]. In fact, BDNF induces and is sufficient for long-term potentiation (LTP) in the hippocampus [18], [67]–[69], a form of synaptic plasticity thought to underlie LTM [5], [35], [37], [70]. It has been shown that BDNF activates several molecular cascades and gene expression pathways; however it is not clear which of the intracellular effectors of this activation are important for memory consolidation.

Here we showed that function-blocking anti-BDNF antibodies infused into the dorsal hippocampus 15 min before or 3 h after IA training, a treatment that impairs formation of avoidance memory, hinder the IA training-induced activation of hippocampal mTOR. We then showed that the two windows of necessity for BDNF differ in their need for new BDNF synthesis. While previously synthesized stored BDNF would be enough around training, new BDNF has to be synthesized about 3 h after training. This finding is consistent with others showing an increase of *BDNF* mRNA in CA1 of the hippocampus 1 h after IA training [36] or within 2–4 hs after application of L-LTP-inducing tetanic stimulation [71]. During training, pre-existing proBDNF could be rapidly cleaved to BDNF by tPA in the absence of *de novo* transcription or translation [18]. Despite other results showing a different effect for pre-training BDNF ASO [72], these differences could be explained by the fact that distinct training procedures and shock intensities induce different molecular activation kinetics [26].

Having established that BDNF is an up-stream activator of mTOR, we then focused our attention on proteins germane to learning, whose translation is regulated by the BDNF/mTOR pathway during plastic processes such as LTP. A likely candidate is the GluR1 subunit of AMPA receptors [73]–[75]. We found that rapamycin and function blocking anti-BDNF antibodies infused into the dorsal hippocampus 15 min before or 2∶45 h after IA training, prevent the rapid increase in GluR1 protein induced by IA training necessary for memory consolidation. Our findings are consistent with others showing that, *in vitro*, BDNF upregulates local translation of PSD95 through mTOR pathway [76] and enhances the expression of GluR1 subunit of AMPA receptors via activation of mTOR [48]. These results are important for two reasons. First, they establish that BDNF is a key molecule that unleashes protein synthesis-dependent memory consolidation in the hippocampus as has been previously proposed [20]. Second, they demonstrate that one of the main effects of BDNF action during memory formation is to increase the expression of GluR1 in synaptic membranes, and that it does so by activating the translation machinery through the engagement of mTOR and its downstream target p70S6K. Although processes other than translation could be associated to the increase in GluR1 expression, so far, the regulation of protein synthesis has been the only mechanism in which mTOR has been implicated. In addition, GluR1 ASO hinders the BDNF/mTOR dependent increase in GluR1 in synaptic plasma membranes and causes a clear-cut LTM deficit 24 h after training. Moreover, we and others have reported that a rapid post-training increase in GluR1 occurs as a consequence of an augmented protein synthesis in addition to translocation form other sub-cellular compartments [33], [48]. Furthermore, it has been shown that dopaminergic stimulation of hippocampal neurons leads to a rapid increase in dendritic expression of GluR1 subunit through a mechanism that requires protein synthesis [77]. It has been shown that *GluR1* mRNA can be transported into dendrites in response to neuronal activity [78], where it can undergo activity-dependent translation at the base of or within spines [79]. Importantly, polyribosomes and other components or regulators of the translational machinery, including mTOR and its downstream targets S6K and 4E-BP have also been reported to be present in spines and dendritic shafts [80], [81]. Alternatively, albeit the rapid increase in GluR1 levels induced by IA training in a synaptic plasma membrane-enriched subcellular fraction may reflect an enhancement in trafficking and membrane insertion of already formed GluR1 subunits [73], [74], it is unlikely that this is the only mechanism, for the afore mentioned reasons. Nevertheless, if this was the case, then our findings could imply that mTOR activation regulates translation of proteins necessary for trafficking and insertion of synaptic receptors. This alternative view deserves further studies.

Together, our findings show that the activated BDNF/mTOR pathway induced expression of GluR1 AMPA receptor subunit in hippocampus synaptic membranes, a key effector protein involved in stabilization of the memory traces is critical for LTM formation.

## Materials and Methods

### Ethics Statement

The experimental protocol for this study followed the guidelines of the USA National Institutes of Health Guide for the Care and Use of Laboratory Animals and was approved by the Animal Care and Use Committees of the University of Buenos Aires.

### Subjects

Male adult Wistar rats (weight, 200–250 g) were housed five to a cage and kept with water and food *ad libitum* under a 12 h light/dark cycle (lights on at 7 A.M.) at a constant temperature of 23°C. Experiments took place during the light phase of the cycle.

### Behavioural Procedures

Animals were allowed to acclimate to the laboratory for 7 days before any experimental manipulation. Inhibitory avoidance was performed as described previously [31]. Briefly, rats were placed on a 5.0 cm high, 8.0 cm wide platform at left of a 50.0×25.0×25.0 cm white acrylic training apparatus, whose floor was a series of parallel 0.2 cm caliber bronze bars spaced 1.0 cm apart. Latency to step down to the grid with the four paws was hand-scored measured. In the training trial the animals received a 0.7 mA, 3 sec scrambled foot shock immediately after stepping-down to the grid. The retention test session was carried out 24 h (LTM) or 7 days after training. This session was procedurally identical to the training session, except that the foot-shock was omitted. All the experiments were realised blinded to the experimental group.

### Surgery and Infusion Procedures

Rats were implanted under thionembutal anesthesia with 22-g guide cannulae in the CA1 region of the dorsal hippocampus at coordinates A−4.3, L±3.0, V−1.4 of the atlas by Paxinos and Watson [82]. The cannulae were fixed to the skull with dental acrylic. Cannulated rats received bilateral intra-CA1 0.5 µl infusions 15 min before or immediately, 1 h, 3 h, 9 h, or 12 h after training. Rapamycin (4.3 pg/side; Cell Signaling, Danvers, MA) was dissolved in 0.1% dimethyl sulfoxide (DMSO). The function-blocking anti-BDNF antibodies (Chemicon, Temecula, CA; AB1513P) were diluted to working concentration (1 µg/µl) with saline. Oligonucleotides (ODN; Genbiotech S.R.L.) were HPLC-purified phosphorothioate end-capped 18-mer sequences, resuspended in sterile saline to a concentration of 2 nmol/ml. Both ODNs were phosphorothioated on the three terminal bases of both 5′ and 3′ ends. This modification results in increased stability and less toxicity of the ODN. BDNF ASO, 5′ -TCTTCCCCTTTTAATGGT- 3′; BDNF MSO, 5′ -ATACTTTCTGTTCTTGCC- 3′. GluR1 ASO, 5′ -TAAGCATCACGTAAGGATC- 3′; GluR1 MSO 5′ - AGCGTATCACAGTATAGAC- 3′. ODN sequences were subjected to a BLAST search on the National Center for Biotechnology Information BLAST server using the Genbank database. BDNF ASO and GluR1 ASO are specific for rat *BDNF* mRNA and *GluR1* mRNA respectively [24], [83]. Control MSO sequences, which included the same number of nucleotides than the ASO but in a scrambled order, did not generate any full matches to identified gene sequences in the database.

6-cyano-7-nitroquinoxaline-2,3-dione (CNQX, 0.5 µg/side; Sigma, St Louis, MO, USA) was dissolved in 2% DMSO The infusion procedure was performed as described previously [36]. Briefly, infusions were in all cases bilateral and had a volume of 0.5 µl, except for ASO and MSO where the volume injected was 1 µl. The entire infusion procedure took around 2 min, including 45 sec for the infusions themselves, first on one side and then on the other, and the handling. Histological examination of cannulae placements was performed as described previously [31]. Briefly, 24 h after the end of the behavioral procedures, 0.8 µl of a solution of 4% methylene blue in saline was infused as indicated above into each implanted site. Animals were killed by decapitation 15 min later and the brains were stored in formalin for histological localization of the infusion sites. Infusions spread with a radius of less than 1.0 mm^3^, as described before [31] and were found to be correct (i.e., within 1.5 mm^3^ of the intended site) in 95% of the animals. Only the behavioral data from animals with the cannulae located in the intended site were included in the final analysis.

### Biochemical Procedures

The animals utilized in the biochemical experiments were divided in three experimental groups: 1) animals trained in the inhibitory avoidance task and killed at different times after training (Trained group, T); 2) animals that received a foot-shock identical to that given to the trained ones but were not submitted to the IA training procedure (the platform was not inside the box, and the animals were put directly over the grid) and killed at the same time points than the trained group (Shocked group, S); and 3) animals withdrawn from their home cages at the same time points than the other two groups and killed immediately thereafter (Naïve group, N); The dorsal hippocampus was dissected out and rapidly homogenized in ice-chilled buffer (20 mM Tris-HCl (pH 7.4), 0.32 M sucrose, 1 mM EDTA, 1 mM EGTA, 1 mM PMSF, 10 µg/ml aprotinin, 15 µg/ml leupeptin, 50 mM NaF and 1 mM sodium) as described previously [36]. Samples of the homogenates (30 µg of protein) were subjected to SDS–PAGE under reducing conditions. After that, proteins were electrotransferred to PVDF membranes which were then blocked and incubated with anti-phospho-mTOR Ser 2448 (1∶2000, Cell Signaling, Danvers, MA), anti-mTOR (1∶2000, Cell Signaling, Danvers, MA), anti-phospho-p70S6K Thr 389 (1∶2000, Cell Signaling, Danvers, MA) and anti-p70S6K (1∶2000, Cell Signaling, Danvers, MA). To analyze the effect of mTOR and BDNF inhibition on GluR1 expression levels, IA-trained rats received bilateral intra-CA1 infusions of rapamycin (4.3 pg/side), anti-BDNF (0.5 ug/side) or saline 15 min before training and were killed by decapitation 15 min thereafter. The hippocampus was dissected out and homogenized as indicated above except that 2 ml of the total homogenate were centrifuged 10 min at 900×g and the supernatant thus obtained was further centrifuged 25 min at 16000×g to obtain a crude sinaptosomal fraction containing the synaptic membranes (Pellet 2; P2) that was resuspended in 300 µl of 20 mM Tris-HCl, pH 7.4, containing 1 mM PMSF, 50 mM NaF and 1 mM sodium orthovanadate. Samples were processed as indicated above and the PVDF membranes were incubated with the following antibodies: anti-GluR1 (1∶1000, Santa Cruz Biotechnology Inc, Santa Cruz, CA); anti-GluR2 (1∶1000, Santa Cruz Biotechnology Inc, Santa Cruz, CA); anti-actin (1∶10000, Santa Cruz Biotechnology Inc, Santa Cruz, CA.)

### Data Analysis

Behavioral and biochemical data were analyzed by one-way ANOVA followed by Newman–Keuls multiple comparison test or Student's *t* test when only two groups where compared.
